# Community perspectives on the implementation of a group psychological intervention for adolescents with depression: A qualitative study in rural Nepal

**DOI:** 10.3389/fpsyt.2022.949251

**Published:** 2022-10-21

**Authors:** Eliz Hassan, Prakash BK, Jananee Magar, Nagendra Luitel, Brandon A. Kohrt, Mark Jordans, Kelly Rose-Clarke

**Affiliations:** ^1^Department of Global Health and Social Medicine, King's College London, London, United Kingdom; ^2^Transcultural Psychosocial Organization Nepal, Kathmandu, Nepal; ^3^Duke Global Health Institute, Duke University, Durham, NC, United States

**Keywords:** Nepal, depression, community, adolescent, mental health

## Abstract

Group-based psychological interventions could help to close the treatment gap for depression in low-resource settings, but implementation barriers exist. In Nepal we sought community members' perspectives on how to implement group interpersonal therapy for adolescents. We conducted qualitative interviews with 25 adolescents with depression (aged 13–18) and seven health and non-governmental organization workers, and four focus groups with non-depressed adolescents, four with parents/guardians, and two with teachers (126 participants total). Data were analyzed using the Framework Method. Participants recommended same-sex groups. School was the preferred location because it is accessible for adolescents and acceptable to parents. Adolescents wanted facilitators from their own community with good communication skills. They did not want parents or teachers to participate in groups but emphasized the need to inform parents and obtain their permission. Community members supported group psychological intervention. School-based psychological interventions facilitated by local people could be an acceptable option in rural Nepal.

## Background

Globally, mental and substance use disorders account for 23% of years lived with disability, and this is predicted to increase two-fold between 2010 and 2030 (1, 2). Adolescence is a period of high-risk for the onset of mental health problems (3). Disorders such as depression and anxiety in adolescence can negatively impact development and lead to social and educational impairment, substance misuse and suicide (4–6).

In low- and middle-income countries (LMICs) a minority of adolescents with mental health problems have access to treatment, even for severe mental disorders (7). Various factors contribute to this treatment gap including inadequate government spending on mental health care, shortage of mental health workers, and discrimination against people with mental health problems which discourages help-seeking (8, 9). Adolescents are more reluctant than any other age group to seek help with their mental health due to difficulties accessing services, concerns about confidentiality, and trust in the provider-patient relationship (10).

To help reduce the mental health treatment gap the World Health Organization (11) developed the mental health Gap Action Programme (mhGAP), which outlines evidence-based packages of care for priority mental disorders in LMIC settings. The mhGAP recommends psychological interventions such as interpersonal therapy (IPT) and cognitive behavioral therapy (CBT) to treat depression and anxiety in children and adolescents. Research shows that these interventions can be delivered by non-specialists (12–14) and that group-based rather than individually delivered therapies may be a cost-effective approach in LMICs (15–17). However, fewer studies have explored the use of psychological interventions for adolescents in LMICs. Among those that have, a variety of interventions have been tested including generic counseling, classroom-based, and trauma-focused treatments (18). Community group interventions such as group IPT show promise for adolescents with common mental disorders, but not in all settings or for all adolescents. For example, in Uganda group IPT for adolescents in internally displaced person camps was beneficial for girls but not boys (12). In South Africa, group IPT did not improve depressive symptoms among orphaned and vulnerable adolescents, and 23% did not attend any group IPT sessions (19). To design and implement more effective and acceptable child and adolescent mental health interventions we need to better understand the needs and preferences of adolescents, parents and other community stakeholders.

The current study was part of a research programme in Nepal that aimed to culturally adapt and evaluate the feasibility of group IPT for adolescents with depression (20). In Nepal, adolescents' experiences of depression are social relationship-orientated and compatible with IPT, and group-based interventions are likely to be beneficial (21). Community group-based approaches have been successfully used with adults and found to be acceptable and preferable (22, 23). In this paper we report findings from the qualitative research component of the adaptation of group IPT where we sought to identify adolescents' and the wider community's perspectives on barriers and facilitators to implementing group IPT.

## Methods

### Setting

We conducted research in Sindhupalchowk, a mountainous district in Nepal. Nepal experienced a 10-year political conflict (1996–2006) and was affected by major earthquakes in 2015 which have impeded efforts to improve health and living standards. Eighty percent of Nepal's population lives in rural areas, but mental health services are mainly concentrated in the cities and there are few age-appropriate services for adolescents (24). A community-based study found a prevalence of 27% for adolescent depression in Nepal (25). Being female, having prior exposure to trauma and being directly affected by the earthquakes are associated with depression (26). Research into the rising rate of adolescent suicide in Nepal has been attributed to academic failure and abuse by parents and teachers (27).

Sindhupalchowk was severely affected by the earthquakes, with marked effects on the mental health of its population. Almost two thirds of adults and 40% of adolescents in the district reported depressive symptoms following the earthquake, which is markedly higher than in other areas of Nepal, or indeed other countries. (26, 28). In Sindhupalchowk health services are limited to one district hospital, three primary health care centers and 76 health care posts (29). Like the rest of Nepal, the population in Sindhupalchowk is hierarchical: Brahman and Chhetri (28%) caste/ethnic groups are the most socioeconomically advantaged; Dalit groups (7%) tend to be the least advantaged. Hinduism (59%) and Buddhism (38%) are the most populous religions (30). The female literacy rate is 51.8% and 67.97% for males (31).

### Interpersonal therapy

The research was conducted as part of the Study to Adapt and Assess the feasibility of group interpersonal therapy in Nepal (SAATHI, meaning friend in Nepali) (20). IPT is a three-phased, psychological therapy originally developed in the US to treat depression among adults and adapted for use among adolescents (32, 33). IPT assumes that mood difficulties are triggered by a person's interpersonal context and relationships. Therapy focuses on addressing one or more interpersonal difficulties related to grief, disputes, life changes and social isolation. Through targeting the interpersonal context and use of the ‘sick role‘, IPT externalizes the problem and aims to instill hope by changing the individual's narrative to one where depression is viewed as treatable. During sessions patients are encouraged to process their emotions, build interpersonal skills, and strengthen protective factors (e.g., social support) to help them better manage problems in stressful times.

### Participants

We sought perspectives from a range of community stakeholders on how group IPT could be optimally implemented for adolescents with depression. Data were transcripts from 25 semi-structured interviews (SSIs) with adolescents with depression aged 13–18 (mean duration 41 min; range 25–67 min); six SSIs with community health workers; and one SSI with a representative from a local non-governmental organization (43 min; 17–65 min). We also conducted ten Focus Group Discussions (FGDs): four with adolescents aged 11-19 (*N* = 38), four with parents/guardians (*N* = 39), and two with teachers (79 min; 40–114 min; *N* = 17). The total number of participants was 126. We purposively sampled adolescents with depression to represent the full range of caste/ethnic groups and ages across genders, including 23 in-school adolescents and two out-of-school adolescents. To recruit in-school adolescents with depression, researchers conducted psychoeducational talks in government secondary schools. They performed role plays using vignettes of depression and asked students to take an information sheet and consent form if they felt the vignette applied to them. Vignettes were informed by the Community Informant Detection Tool (CIDT) developed as a tool for pro-active case detection in Nepal (34). Researchers assessed adolescents who returned signed consent forms using the Depression Self-Rating Scale (DSRS) and Child Function Impairment Tool (adapted for adolescents in a rural setting) (35). Adolescents scoring 14 or above on the DSRS (sensitivity 0.71, specificity 0.81) and four or above on the functional impairment tool were invited to an interview. Most interviews were conducted in school, during or after class, in a quiet and private room away from other people. Most interviews with out of school adolescents were conducted in local health offices, schools, or neighbors' homes.

To provide alternative perspectives on how the intervention should look we conducted FGDs with teachers and adolescents (who were not assessed for depression) from the same schools. Researchers invited teachers to participate in these FGDs and to nominate appropriate adolescent participants. We recruited out-of-school adolescents, parents, health workers and the NGO representative through our contacts in the local community.

SSI and FGD topic guides were divided into two parts. The first part included questions to explore direct and indirect experiences of depression, coping strategies and existing and desirable forms of help and support. In the second part, we described group IPT and asked about acceptability, suitability and relevance, how the intervention should be delivered (e.g., group size and composition, venue, day and time, possible facilitators), potential barriers to participation, and how to promote engagement. We used the term *man ko samasya* (meaning heart-mind problem) to enquire about depression as this has been found to be a non-stigmatizing way to communicate about depression in community settings (36). SSIs and FGDs were conducted in Nepali by the project coordinator (male, MA Sociology) and senior research associate (female, MA Sociology). Both are experienced Nepali qualitative researchers and were aided by two trained Nepali research assistants. RAs conducted SSIs and FGDs in school spaces, health posts and other community locations.

### Ethical consideration

The study was approved by the Nepal Health Research Council (637/2018) and King's College London Research Ethics Committee (HR-18/19-8427). We sought permission from local government authorities and the principals of participating schools. We obtained informed consent for participants and explained the reasons for the research. For participants aged 17 and under we also obtained consent from their parent or guardian.

### Data analysis

Supplementary Table 1 presents the completed COREQ (37) checklist of items to report in a qualitative study. SSIs and FGDs were audio-recorded, transcribed, and translated into English. NPL and PBK checked the quality of the transcriptions. PBK, EH and KRC analyzed the data using the Framework Method (38). We chose this approach because it is suitable for managing large datasets and because it is ideal in situations where multiple researchers are involved in data analysis. We initially developed a deductive analytical framework based on the questions in the topic guide. PBK and EH coded 10 transcripts in parallel to check consistency in application. They also refined the framework by incorporating new inductive codes and organizing the codes into broader themes. To re-check consistency EH and KRC coded a further three transcripts using the revised coding framework. The remaining transcripts were divided between the three researchers for coding with the revised framework. Data were charted into a coding matrix in Microsoft Excel to identify patterns between and across codes and participants. Attention was given to negative cases to recognize alternative viewpoints and increase the validity of the findings. Once the transcripts were analyzed EH reviewed the data for trends and patterns, and findings were reviewed by KRC and PBK.

## Findings

Table 1 presents characteristics of the study participants. Adolescents with depression had a mean DSRS score of 19 (female median 20, range 14–29; male median 18, range 14–32), 56% were female, and the median age was 15 (range 13–18).

**Table 1:** Participant characteristics.

	**Adolescent SSI *n = 25***	**Adult SSI *n = 7***	**Adolescent FGD's *Four groups (n = 38)***	**Parent FGD's *Four groups (n = 39)***	**Teacher FGD's *Two groups (n = 17)***
Gender					
Female	14 (56%)	4 (57%)	17 (50%)	19 (50%)	*Mixed*
Male	11 (44%)	3 (43%)	21 (50%)	20 (50%)	
Age					
Mean age (SD)	15.4 (1.5)				
Median age	15				
Age range	13–18		11–19	21–66	26–57
Caste/ethnicity					
Brahman/Chhetri	13 (52%)	4 (57%)	*Mixed*	*Mothers: mixed* *Fathers: Brahman/Chhetri*	*Mixed*
Janajati	7 (28%)	2 (29%)			
Dalit	5 (20%)				
Madhesi		1 (14%)			

SSI, semi-structured interview; FGD, focus group discussion; SD, standard deviation.

We identified six major themes: (i) adolescents need emotional support; (ii) the importance of finding a convenient time and place for group sessions; (iii) group-based therapy facilitates peer support; (iv) group facilitators must understand the local context; (v) advantages and disadvantages of mixing adolescents with different experiences; (vi) the need to increase buy-in from parents. Table 2 summarizes the key findings and implications for group-based psychological interventions.

**Table 2:** Table summarizing key findings.

**Themes**	**Description of theme**	**Implications for group-based psychological interventions**
Adolescents need emotional support	Emotional distress and heart-mind problems recognized as common among adolescents. Greater awareness and support needed.	• Provide psychoeducation in the community to increase awareness of adolescent mental health problems • Promote adolescent engagement in therapy through games, music, and sport
The importance of finding a convenient time and place for group sessions	Location needs to be easily accessible, and the timing of groups should not interfere with school and household work	• Supports a school setting • A quiet, private space away from other students is preferable • Consider holding classes in the early morning before school, at lunchtime break, or on Saturdays
Group-based therapy facilitates peer support	A group-based approach facilitates problem sharing and solving between adolescents.	• Stress to the importance of maintaining confidentiality to the group • High risk adolescents should be identified and individual therapy considered
Group facilitators must understand the local context	Facilitators need to be local so that they understand the culture and are accessible to adolescents in the community	• Facilitator caste, role, and gender should be decided based on the composition of the group • Adolescents from Dalit and Janajati groups may feel uncomfortable with a Brahman/Chhetri facilitator • Teachers are not suitable facilitators because they can be unapproachable, judgemental and discriminating against students with mental health problems.
Advantages and disadvantages of mixing adolescents with different experiences	Adolescents feel more comfortable in groups with their friends but recognize the opportunity to learn from adolescents with different backgrounds and experiences	• Single-sex groups are preferable. • Consider effects of caste-based discrimination on participation
The need to increase buy-in from parents	Concern that parents would not allow adolescents to attend due to stigma and conflict with other responsibilities. Parental permission deemed necessary to ensure adolescent attendance and consistent engagement.	• Facilitators need to be flexible in adapting to family concerns and unexpected absences • Increased communication between facilitators and parents, and meetings with parents and adolescents to address parents' concern and obtain permission

### Adolescents need emotional support

Participants expressed the need for community-based support for adolescents with heart-mind problems, and were generally positive about a psychological intervention.

“*I think it would be nice. Everyone would share their problem with each other and so everyone would know about each other. They would say, “This person has a similar problem to me. They have already gone through this.” […] They might suggest and provide possible solutions to the problems. That way I would feel light and relaxed.”* (Male aged 18)

Parents, teachers and health workers thought adolescents needed the opportunity to “express their emotions” to alleviate their depressive symptoms. One health worker had found that counseling was effective for patients and that sharing and normalizing their experience increased “hope for life.” Parents had mixed views about community-based programmes and described how previous social initiatives had failed to bring about long-term change.

Participants perceived that recruiting adolescents to a psychological intervention would be challenging. Teachers warned that an intervention would fail if adolescents were not self-motivated to access help and share their heart-mind problems. Participants stressed the need to increase awareness of mental health problems and encourage adolescents to seek help. They suggested incorporating stories, games, music, and sport into group sessions to promote engagement.

### The importance of finding a convenient time and place for group sessions

Participants unanimously agreed that the location and timing of the intervention would determine its success. Groups should be held somewhere familiar to the adolescents and close to their homes. They identified geographical remoteness of communities and lack of transport as barriers to attendance, and suggested adolescents would be willing to walk 30-min to 2 h to reach the groups.

Adolescents and parents felt groups should be in school for several reasons. First, adolescents and health workers thought that it would be less stigmatizing to attend groups in school rather than in a health facility. Second, parents said they would be more likely to trust and permit their child to attend an intervention that took place in schools rather than in the community. Third, private spaces were generally available in schools. Last, adolescents were already attending school during the week, and it would therefore be a convenient location. However, some adolescents thought that out-of-school or married adolescents may prefer a community building or health post.

There were mixed views about the timing of sessions. Adolescents, teachers and parents did not want sessions to interfere with schoolwork, especially exam preparation, but recognized the difficulties of attending sessions out of school time.

“*There would be lots of obstacles [to me attending a group on Saturdays]. I only get to stay [at home] one day in a week and in that single day, I have to wash clothes, take a bath, clean my home. They take my whole day. Sometimes I have to load bricks. I need to work the whole of Saturday.”* (Female, aged 15)

### Group-based therapy facilitates peer support

Parents and health workers preferred the concept of group rather than individual sessions because group sessions might help to alleviate adolescents' loneliness. Fifteen of the 25 adolescents with depression said they would feel comfortable in a group setting and that listening to others with similar problems would make them feel “happier” and “light”.

“*I think the programme would be good because […] adolescents would get a chance to open up to someone. They would also get a chance to present their future plans in front of all those people. They would be able to express their thoughts, positive thoughts with all the courage.”* (Female, aged 15)

Five adolescents were concerned about confidentiality in a group and being teased by others. Only one adolescent preferred individual sessions:

“*[Other group members] would know me well and I cannot share in front of people who know me. They might circulate my problems with friends in this community and I would feel very bad if that happens. They might think badly of me and might also judge me for what I am.”* (Male, aged 18).

Health workers suggested individual sessions might be appropriate for adolescents with more “difficult” problems such as suicidal thoughts, but that it would be unlikely there would be enough facilitators to offer them to everyone.

### Group facilitators must understand the local context

We asked participants about desirable characteristics of an intervention facilitator and probed about the importance of their caste/ethnic group. Twenty out of 25 adolescents with depression said that caste was not important if the facilitators treated adolescents equally, with ‘respect’ and empathy. Parents, teachers, and health workers, most of whom were from Brahman/Chhetri groups, did not perceive caste as an issue. Adolescents from more marginalized groups (Dalit and Janajati groups) had contrasting views.

“*Most of the people in this community belong to a “higher caste.” They still practice discrimination and untouchability based on caste. Even if they listen to my problem they would judge based on my caste identity, interpret my problem wrongly, and wouldn't understand my problem.”* (Male, aged 18)

Some participants thought the facilitator's religion was also relevant.

“*We have some active Christian brothers [in our community]. […] If they distribute something then people will take it negatively, saying that they were doing so to promote their religion.” (Teacher)*

Eleven out of 25 adolescents said facilitators would be better able to empathize with participants of the same gender, and participants would be more comfortable sharing. A minority of adolescents and some parents thought it could be advantageous to have male facilitators.

“*It is better to have a female [facilitator] for girls but how I also understand it is that it will be effective and trustworthy if a male would speak. For example, if I go and tell something in my house, my children will not obey it but if [a male] goes and tells the same thing it will be okay. There lies a difference between male and female and still it exists. If female says something they say that females shout but if male says something it seems as reality. Similarly, females cannot put forward their issues in front of males and there lies the problem*.” (Mother)

Adolescents had mixed views about the acceptability of teachers as facilitators. Twelve out of 25 adolescents with depression were in favor because teachers were perceived as “educated” and “guardians” who could “help [adolescents] learn and understand about their heart-mind problems.” However, nine adolescents with depression, and adolescents in three out of four FGDs said they would feel shameful sharing their heart-mind problems with teachers. Moreover, teachers could be strict, unapproachable, and perpetrators of violence against adolescents.

Participants recommended recruiting facilitators locally because they would understand the context and be more accessible to adolescents. However, participants also perceived greater risks to confidentiality with a facilitator from their own community. Some adolescents suggested nurses and social mobilisers (adults with a higher secondary education, recruited from the community to help implement government development initiatives) as potential facilitators.

### Advantages and disadvantages of mixing adolescents with different experiences

Most participants suggested a group size of between 6 and 10 adolescents because this would be sufficient to stimulate discussion and give everyone the opportunity to speak. Larger groups were perceived to be too noisy and disorderly, and unsuitable for sharing heart-mind problems.

Adolescents preferred to be in a group with their friends.

“*If friends are kept together they would support me. Sometimes if I speak wrong, they would correct me. I would be courageous if I am with friends.”* (Male, aged 14)

Parents wanted to be involved in the groups to help their children and better understand mental health. However, most (15/20) adolescents did not support parental involvement in groups due to fear of being reprimanded.

“*Though the family member would not do anything in the session, he/she might do something when [the adolescent] reaches home. He/she may beat, hit, or threaten [the adolescent] […] Nothing should be told to their family member […]. If [we] say [in groups] what is on our mind, the issue will be known to everyone in the village. The villagers might backbite and due to that a big fight can occur […]. Therefore, my opinion is not to include family members.”* (Male aged 17)

FGD participants, 18 depressed adolescents and five health workers recommended single sex groups because of the different challenges and stressors faced by males and females. Moreover, male adolescents said that they would be uncomfortable sharing their feelings in front of girls and female adolescents were worried that males would tease and laugh at them.

Around half the participants preferred separate groups for younger (13 to 14 years) and older adolescents (15 to 19 years). This was because of concerns that younger adolescents would be too nervous to open-up in front of older adolescents, and because younger and older adolescents were perceived to be at different stages of life with different interpersonal problems. Seven adolescents recommended mixed age groups so that older adolescents could advise the younger ones.

Parents and health workers thought it was important for groups to comprise adolescents with similar problems who could understand each other; they did not support inclusion of school-going and non-school-going and married and unmarried adolescents in the same group. However, school-going adolescents thought it could be an opportunity to learn from their peers.

“*I think [married adolescents] could be mixed together with us. We can understand the situation from both perspectives. We can learn things from married adolescents. We can also know about the consequences of getting married in early age and being separated. We can also learn from them that no one should get married that early and should not marry an unknown person.” (*Female, aged 15)

Most adolescents supported mixed caste/ethnicity groups on the basis that they already mixed at school. Adolescents from “high caste” groups said they did not discriminate against Dalit groups but their grandparents and parents did and may stop them from participating.

### The need to increase buy-in from parents

Participants were concerned parents would not allow adolescents to participate in groups for fear of being stigmatized.

“*The thing is how the parents will react when their children are labeled as having heart-mind diseases. […] Due to fearing that no one will marry their daughter they will not want to expose such problems…”* (Teacher)

A health worker highlighted the importance of using non-stigmatizing terms to describe the intervention and to avoid group members being labeled as “pagaal” (meaning mad). Adolescents described how they had to prioritize housework and paid work to support their families and avoid punishment. If they missed a session facilitators would need to be flexible and understanding. Over half the participants (13 adolescents with depression, five health workers, and in five of the FGDs) suggested providing incentives such as lunch, money or skills training to promote attendance.

All participants emphasized the need for parental permission. Parents and adolescents viewed informing parents about the programme content and location and gaining their permission as essential. However, adolescents thought it would be more empowering and motivating if the facilitator communicated directly with them, rather than through their parents.

## Discussion

We explored adolescents', parents', teachers' and health-workers' perspectives on how to implement group IPT for adolescents in rural Nepal. The study gives voice to the local community and makes a valuable contribution to the scant literature on adolescent psychological interventions in LMICs. Participants perceived adolescents need emotional support to improve their heart-mind problems and group IPT could be helpful. They favored a group-based approach within a school setting so adolescents can support each other. Although participants had mixed views on the ideal caste/ethnicity, gender and religion of a facilitator, they agreed it should be someone familiar with the local context but not a teacher. Some participants favored mixing younger/older, in/out of school, and married/unmarried adolescents to facilitate peer to peer learning, whereas others were concerned that adolescents in mixed groups would be unable to relate to each other. Adolescents preferred not to involve parents and teachers in groups due to concerns about confidentiality and being reprimanded. They described how they had to prioritize household and paid work so finding a convenient time and location for groups was important.

The education system is an increasingly popular delivery platform for child and adolescent mental health care in LMICs (13, 39, 40). There are several reasons for this. First, schools are potentially the most accessible option because a large proportion of adolescents attend school and there are often more schools than health facilities in LMIC settings. Second, interventions could draw on existing school resources (personnel, space) and be scaled up through the education system, which could be a cost-effective and sustainable approach. Third, in some contexts schools are already delivering nutrition and WASH (Water, Sanitation and Hygiene) programmes and could theoretically be expanded to incorporate mental health. Fourth, interventions vetted by schools are likely to be more acceptable to parents and adolescents. However, a drawback of implementing interventions in school is that there may be limited time and space for extracurricular activities, particularly during busy exam periods. Private and quiet rooms for therapy may not exist. Moreover, schools can be a dangerous place for adolescents exposed to physical, emotional and sexual abuse by teachers and students. In our study some adolescents had poor relationships with teachers, and teachers had stigmatizing views on mental health, suggesting they would not be appropriate IPT facilitators. In a meta-analysis of school-based interventions for adolescent depression and anxiety, it was found that interventions delivered by internal school staff did not improve symptoms compared to controls (41). Moreover, school-based intervention may exclude the most vulnerable adolescents, including out-of-school and married adolescents. A recent review of implementation outcomes and strategies for depression in LMICs found that existing research has focused mainly on intervention acceptability, appropriateness, and feasibility (42). More research on the cost, scalability and sustainability of school-based approaches in LMICs is urgently needed.

In our study, participants were concerned about the stigma of participating in a psychological intervention and how language can exacerbate or mitigate this. Nepali ethnopsychological models of the self comprise various components including *man* (heart-mind) and *dimaag* (brain-mind). Interventions that focus on mental disorders and dimaag are highly stigmatized, whereas those that link symptoms to the *man* and adopt a psychosocial framework are more acceptable and show good engagement among adolescents (36, 43, 44). Anti-stigma training to address assumptions about violent tendencies among people with mental illness and to challenge views that mental illness cannot be treated improved clinical behavior among Nepali health practitioners (45). Beyond Nepal, various anti-stigma methods have been tested including lectures, campaigns, films, printed material, workshops, CBT, and social contact between people with and without mental health problems (46, 47). Stepped care interventions for depression that combine treatment with a universal anti-stigma component are a promising approach and have the potential to increase mental health literacy and demand for mental health care, as well as reducing depression. One such example is the PRemIum for aDolEscents (PRIDE) programme in India, which combines a brief lay counselor-delivered problem-solving intervention with whole-school and classroom-based sensitization activities including a brief animation and group discussions (13).

It remains unclear if or how parents should be involved in adolescent psychological interventions. A recent meta-analysis of school-based interventions for depression showed that interventions not involving parents were beneficial compared to controls, whereas those that involved parents had no effect (41). Others support the involvement of parents (48). In existing IPT interventions, the extent of parental involvement depends on the child's developmental stage. For example, in Family Based IPT for pre-adolescents aged 8–12, parents attend dyadic sessions with their child to learn communication and problem-solving skills, and strategies to deal with conflict (49). In IPT for adolescents aged 12–18 there is less parental involvement, reflecting adolescents' increased desire for autonomy and capacity for abstract thinking (50). Parents are involved in initial sessions to help gather information about the adolescent's depressive symptoms and formulate appropriate therapeutic goals. They are also invited to attend a session in the middle and terminal phases to review their child's progress and contribute to decisions about treatment (51). Adolescents in our study did not want to involve parents in groups but recognized the need to obtain their permission. To increase parent buy-in and reduce stigma, other studies implemented pre-screening briefings with parents (52), ran joint structured activities (53), and created School Health Promotion Committees with parent representatives (54). Future research is needed to directly compare interventions with and without parental involvement and identify the most effective approaches.

Our findings suggest the importance of considering the broader socio-economic context of adolescent psychological interventions. Many adolescents in Nepal have paid and unpaid work commitments and caring responsibilities which are vital to their household. Finding a convenient time and place to conduct sessions could be difficult. Alternatives to in-person therapy such as phone-based or online sessions may be more acceptable, especially considering recent COVID-19 national lockdown policies (55, 56). Monetary incentives and cash-transfer programmes could also help to reduce financial pressure on adolescents though may compromise intervention cost-effectiveness (57).

Strengths of our study include the relatively large and diverse sample of adolescents, teachers, parents and health workers, and triangulation of data from SSIs and FGDs. Our findings are relevant to other communities in Nepal although because we were only able to include two out-of-school adolescents they may not be generalizable to out-of-school populations. High-caste respondents were over-represented in our sample and we were unable to adequately capture the perspectives of marginalized groups such as Dalits and Janajatis who are disproportionately impacted by mental health problems (58). We focused on community perceptions of how to optimally *implement* a group psychological intervention rather than how to adapt its therapeutic content. We describe our approach to cultural adaptation of group IPT elsewhere (20) and future research could explore community preferences for different psychological models. We used a systematic, transparent analysis procedure with a paper trail to ensure our findings are dependable and confirmable. We cannot exclude the possibility of social desirability bias because participants knew that researchers were involved in adapting and implementing group IPT in Sindhupalchowk.

## Conclusion

Community members supported the notion of a group-based psychological intervention for adolescents with depression. Adolescents preferred the intervention to be facilitated by local people but not teachers, and to limit involvement of their parents. Our findings inform implementation in other low-resource settings by outlining a potential role for community-level anti-stigma activities and the need for more research on parental involvement in adolescent psychological interventions. Implementation research is also needed to identify how to promote the cost-effectiveness, sustainability and scalability of IPT through the education system.

## Data availability statement

Due to the sensitive nature of the topic of this study and difficulties fully anonymising the transcripts, the data is not suitable for sharing.

## Ethics statement

The studies involving human participants were reviewed and approved by the Nepal Health Research Council and King's College London Research Ethics Committee. Written informed consent to participate in this study was provided by the participants' legal guardian/next of kin.

## Author contributions

MJ and KR-C conceptualized the study. PBK and JM supervised and participated in data collection. EH, PBK, and KR-C conducted the analysis. EH wrote the first draft of the paper. All authors were involved in developing the analytical framework. All authors reviewed the paper and approved the final version for submission.

## Funding

The study was co-funded by the UK Medical Research Council, the National Institute for Health Research and the Department for International Development (study reference: MR/R020434/1). The funders had no role in the collection, analysis, and interpretation of data; in the writing of the articles; and in the decision to submit it for publication.

## Conflict of interest

The authors declare that the research was conducted in the absence of any commercial or financial relationships that could be construed as a potential conflict of interest.

## Publisher's note

All claims expressed in this article are solely those of the authors and do not necessarily represent those of their affiliated organizations, or those of the publisher, the editors and the reviewers. Any product that may be evaluated in this article, or claim that may be made by its manufacturer, is not guaranteed or endorsed by the publisher.
